# Optical Coherence Tomography Study of Choroidal Response to Exercise-Induced Hypertension in Chronic Central Serous Chorioretinopathy

**DOI:** 10.3390/jcm13216580

**Published:** 2024-11-01

**Authors:** Anindya Samanta, Giulia Gregori, Alessio Muzi, Ramkailash Gujar, Cesare Mariotti, Daniela Fruttini, Kiran K. Vupparaboina, Jay Chhablani, Massimo Nicolò, Chiara M. Eandi, Felice Cardillo Piccolino, Marco Lupidi

**Affiliations:** 1 Department of Ophthalmology, Baylor College of Medicine, Lubbock, TX 77030, USA; 2 Eye Clinic, Department of Experimental and Clinical Medicine, Polytechnic University of Marche, 60020 Ancona, Italy; g.gregori98@gmail.com (G.G.);; 3 Eye Clinic, Humanitas-Gradenigo Hospital, 10153 Torino, Italy; 4 Cornea and Stem Cells Department, Dr. Shroff’s Charity Eye Hospital, Daryaganj, New Delhi 110002, India; 5 Department of Medicine and Surgery, Section of Internal Medicine, University of Perugia, S. Maria Della Misericordia Hospital, 06156 Perugia, Italy; 6 UPMC Eye Center, University of Pittsburgh, Pittsburgh, PA 15260, USAjay.chhablani@gmail.com (J.C.); 7 Fondazione Italiana Macula ETS, Di.N.O.G.Mi., University Eye Clinic, Viale Benedetto XV 5, 16132 Genova, Italy

**Keywords:** central serous chorioretinopathy, optical coherence tomography, choroidal vascularity index, mean ocular perfusion pressure, hemodynamic stress, physiological mechanisms regulating the choroidal blood flow

## Abstract

**Background/Objectives**: The aim of this study was to evaluate the choroidal vascular response using optical coherence tomography (OCT) in patients with chronic central serous chorioretinopathy (CSCR) during transient increases in blood pressure. **Methods**: This observational, case–control study enrolled chronic CSCR patients and age-matched healthy controls. OCT scans of the macula were performed at rest and during hand-grip (HG) isometric exercise. Mean ocular perfusion pressure (MOPP) and subfoveal choroidal thickness (SCT) were measured at baseline and during stress. Quantitative OCT assessment included the bright area (BA, stroma), dark area (DA, vascular lumen), and total choroidal area (CA). The choroidal vascularity index (CVI) was calculated as DA/CA to assess vascular response to stress. A comparative analysis between CSCR patients and controls was conducted. **Conclusions**: MOPP was significantly higher (*p* = 0.008) at baseline in CSCR patients and further increased under stress compared to controls. SCT and CA were both significantly higher in CSCR patients than in healthy subjects at rest and under stress (*p* < 0.001), but no change occurred after HG. A significant decrease in CVI (*p* = 0.005) was noted in controls under stress, but not in CSCR patients. Additionally, a negative correlation between CVI and MOPP was found in healthy subjects (−0.648; *p* = 0.043). The study demonstrated a choroidal vasoconstrictive response to stress in healthy subjects, as evidenced by a decrease in CVI, but not in CSCR patients. This suggests that CSCR patients may experience impaired choroidal blood flow regulation, resulting in potentially higher perfusion pressures during stress without compensatory vasoconstriction, potentially affecting the choriocapillaris.

## 1. Introduction

Central serous chorioretinopathy (CSCR) is a self-limiting disorder with fluctuating idiopathic retinal detachments of the posterior pole. However, it may remit with focal retinal neurosensory detachments that can result in symptomatic visual decrease secondary to outer retinal atrophy [1,2]. In chronic CSCR, prolonged accumulation of subretinal fluid (SRF) can result in permanent visual deterioration if left untreated [3,4,5,6,7].

Although the exact molecular mechanisms of CSCR have remained uncertain, the choroid seems to have a key role in the pathophysiology of CSCR [8]. Indeed, choroidal vascular hyperpermeability and choroidal thickening are classic CSCR hallmarks [9,10,11]. Moreover, ICGA studies have shown vascular filling delays and venous congestion due to abnormal choroidal blood flow in CSCR patients [12,13]. The enhanced depth imaging and swept-source optical coherence tomography (OCT) have identified dilated large choroidal vessels, showing chronic changes in the choroidal circulation in CSCR patients [14,15].

The choroid regulates blood flow through an autonomic nervous system and inner autoregulatory mechanisms [16,17,18,19]. It can rapidly regulate the vessels during sudden variations in the mean ocular perfusion pressure (MOPP), as evidenced by previous studies with changes to the blood pressure (BP) or intraocular pressure (IOP) [20,21,22]. In contrast, in chronic CSCR patients, the subfoveal choroid is unable to respond to a sudden increase in blood flow provoked by increases in MOPP and BP [23]. Therefore, the CSCR pathogenesis might be significantly influenced by this choroidal vascular dysregulation; this local choroidal hemodynamic impairment in CSCR needs to be further characterized. Moreover, the impact of BP changes on choroidal perfusion should also be evaluated, since hypertension is often associated with CSCR [24,25].

In the present study, we have examined the choroidal vascular response of patients with CSCR to an abrupt MOPP increase, induced by an isometric exercise, using spectral domain OCT (SD-OCT). This real-time quantitative evaluation of the choroidal tissue showed that a dysfunctional choroid is present in patients with CSCR [26].

## 2. Materials and Methods

### 2.1. Design

This case–control study was conducted at the Eye Clinic, S. Maria Della Misericordia Hospital (Perugia, Italy), and at the Fondazione Italiana Macula ETS (Genova, Italy). CSCR patients and healthy controls were examined by SD-OCT before and during the isometric exercise involving a hand-grip test (HGT) to assess how the choroidal vasculature would be affected by an induced rise in MOPP [27,28,29]. Informed consent was gained from all subjects enrolled in the study. The local Ethics Committees (Ancona and Genova IECs) approved the study that adhered to the 1975 Helsinki Declaration and its later amendments.

### 2.2. Population

Patients with a confirmed diagnosis of chronic CSCR and age-matched healthy subjects were consecutively enrolled between December 2022 and December 2023. Chronic CSCR was defined as the presence of symptoms for more than 6 months. Eyes showing any amount of subretinal fluid accumulation, fibrovascular pigment epithelium detachments, or other retinal abnormalities in the central macula that might compromise the structural-OCT imaging assessment of choroidal layer were excluded from the study. Patients showing serous retinal detachment/s outside the OCT scanning area at the time of the examination were considered eligible for the study. In the case of bilateral chronic CSCR, with both eyes eligible for the study, the study eye was selected in a random fashion. For safety reasons, those CSCR patients being treated for systemic hypertension (defined as systolic BP higher than 150 mm Hg or diastolic BP higher than 90 mm Hg) were excluded in the study. Additional exclusion criteria included a refractive error of 3 diopters or more, a confirmed diagnosis of glaucoma, any prior ocular surgery, laser photocoagulation or photodynamic therapy (PDT) within the last 12 months, systemic steroid use in the previous 6 months, or any evidence of eye disease that could impact the study’s purpose.

In the control group, eligible subjects were considered those with best-corrected visual acuity (BCVA) of 0 LogMAR or higher and no history or actual evidence of retinal or relevant systemic diseases including hypertension.

### 2.3. Experimental Protocol and Imaging

At baseline, both patients and controls underwent BP and IOP measurement, BCVA testing, slit lamp biomicroscopy, fundus evaluation, and macular structural SD-OCT performed in mydriasis (obtained by Tropicamide 1%, Visumidriatic, Visufarma, Italy). The HGT experimental protocol, following the American Society of Hand Therapist recommendations, has been previously described by our group. Baseline OCT was set as reference scan using the Spectralis SD-OCT certified follow-up system to maximize the standardization of the examinations. The second structural OCT was acquired 1.5 min after the beginning of the HGT as a follow-up scan per the protocols in previous studies [2,30,31]. The BP and IOP were recorded before each OCT scan [32]. All the structural OCT scans were acquired by the Spectralis OCT, an 85 kHz SD-OCT device (Heidelberg Engineering, Heidelberg, Germany). For our purpose, a high-resolution structural OCT volume scan of 20° × 20° (6 × 6 mm approximately) centered in foveal area was carried out in each enrolled subject. The volume scan was conducted using 97 B-scans and the automated averaging process was set at 30 frames per scan (inter B-scan distance: 60 µm). The two OCT imaging sessions (baseline and during HGT) were performed by the same experienced operator (R.G.), during a single visit, between 9 and 11 AM to minimize diurnal variation in choroidal thickness [33]. All scans were independently assessed by two investigators (F.C.P. and M.L.) to evaluate the image quality for the quantitative assessment (minimal required quality index [QI] of 30 dB).

### 2.4. Choroidal Quantitative Assessment

The choroidal area (CA), the subfoveal choroidal thickness (SCT), and the choroidal vascularity index (CVI), an OCT parameter for measuring the vasculature status of the choroid, were computed as per our previous publications using automated segmentation and binarization algorithms [34,35]. CVI was calculated as the ratio of the luminal area to the total choroidal area; a high CVI represents dilation of the choroid vessels while a low CVI represents constriction of the choroid vessels. In brief, automated binarization of the OCT B-scan (both horizontal and vertical) was carried out followed by automated segmentation of the binarized choroid, including the choroidal stromal and vasculature layer analysis.

### 2.5. Statistical Analysis

The absolute and relative differences and the coefficient mean and standard deviation for the groups of patients analyzed (CSCR and controls) are presented in the tables. In some cases, the data are represented graphically. The Kolmogorov–Smirnov test determined that the data were non-parametric. Based on the result obtained, the Wilcoxon test for paired data was used to see the variability between baseline and stress test. The Spearman coefficient was used to calculate the correlation between the analyzed variables. The comparison between the two groups (CSCR and controls) with and without HG was made using the non-parametric test of Wilcoxon–Mann–Whitney. Results where *p* < 0.05 were considered significant. The processing was carried out with SPSS 25 software for Windows (IBM corp. IBM, Armonk, NY, USA).

## 3. Results

### 3.1. Study Population Ocular and Hemodynamic Data

A total of 30 eyes of 30 patients with chronic CSCR and 30 eyes of 30 age-matched healthy subjects were enrolled in the study. There were 23 males and 7 females in each group. The mean age of CSCR patients was 47.4 ± 7.4 years (range: 33 years to 65 years) and the mean age for controls was 46.1 ± 7.0 years (range: 37 years to 59 years). The ethnicity of the patients in both groups was Caucasian. The mean IOP values were 13.4 ± 1.7 mmHg in the CSCR group and 14.4 ± 1.5 mmHg in the control group. None of the control subjects reported any current or previous systemic therapy. No adverse events were noted during the study. All the recruited subjects tolerated the simultaneous structural OCT assessment and the HGT well.

**Figure 1:**
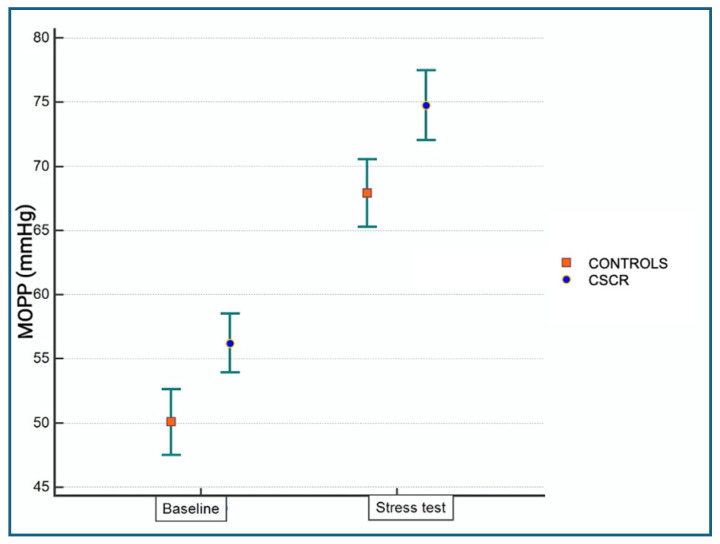
Mean value and confidence interval of the MOPP in CSCR patients and healthy controls both at baseline and under stress. Image shows the significant difference between CSCR patients and healthy controls both at baseline and under stress when *p* < 0.05.

**Figure 2:**
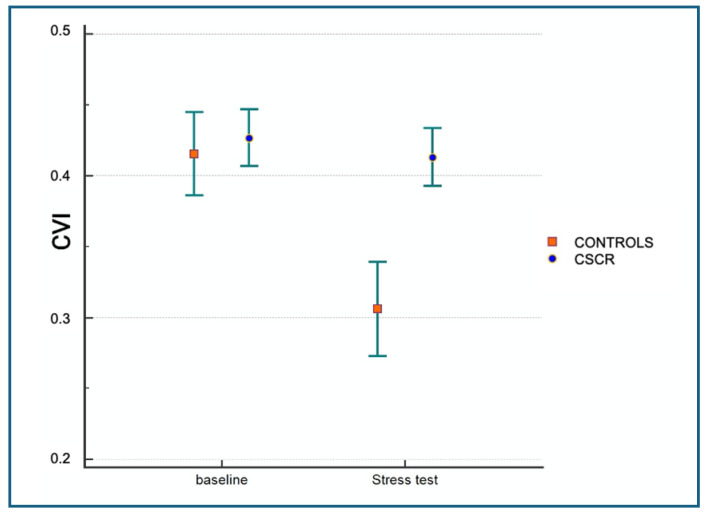
Mean value and confidence interval of the CVI in CSCR patients and healthy controls both at baseline and under stress. Image shows that there is a statistically significant variation in the CVI in healthy controls before and after the stress test when *p* < 0.05. In CSCR patients, no significant CVI variation is shown between baseline and HGT data when *p* > 0.05.

MOPP was calculated using the diastolic BP (DBP) and systolic BP (SBP) by the formula MOPP = 2/3 [DBP + 1/3(SBP − DBP)] − IOP [36]. The mean and standard deviations of the MOPP at baseline and during the isometric exercise are shown in Table 1 and Table 2. Both the CSCR and control groups showed a statistically significant increase in MOPP (*p* < 0.05) between the baseline and HGT levels. Baseline and under-stress data were significantly higher (*p* < 0.05) in CSCR patients compared to healthy subjects (Figure 1). The baseline and under-stress values of the OPP of CSCR patients did not show a significant difference in absolute or relative terms (∆ and ∆r) when compared to the values of healthy controls. (Figure 2).

### 3.2. Quantitative OCT Assessment

**Figure 3:**
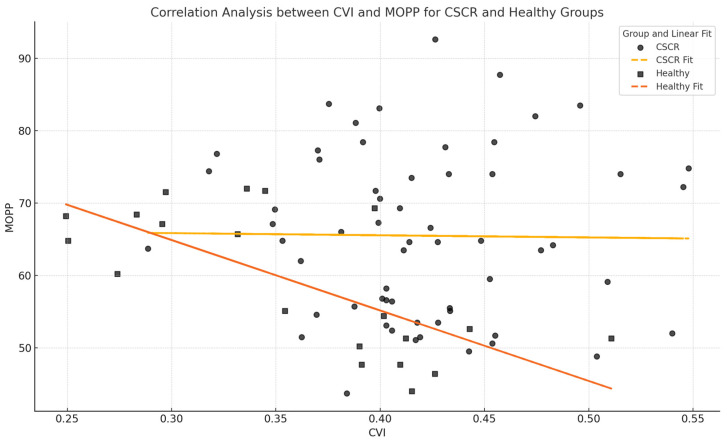
The scatter plot illustrates the trends between CVI and MOPP. The combined correlation for all groups is r = −0.07, with a *p*-value = 0.54 indicating no statistically significant relationship between CVI and MOPP across the dataset. This suggests that there is no significant linear association between CVI and MOPP for either CSCR patients or healthy controls under the conditions studied.

The results of the quantitative evaluation of the CA, SCT, and CVI are shown in Table 1 and Table 2. A statistically significant difference (*p* < 0.05) between baseline and HGT values in CVI was shown in the control group but not in patients with CSCR. CVI data at baseline did not show a statistically significant difference in the two groups; the data after HGT were significantly different (*p* < 0.05) between the two groups. Similarly, compared to CSCR subjects, healthy subjects had higher absolute and relative CVI between baseline and following HG (Table 2). Spearman’s correlation did not show a significant linear correlation between MOPP and CVI variations (Δ) for either CSCR patients or healthy controls (r = −0.07, with a *p*-value = 0.54) (Figure 3).

## 4. Discussion

In this study, SD-OCT was used to show the choroid vessels’ response to increases in MOPP. SD-OCT performed at baseline and then after HG, which was to simulate abrupt transient increases in MOPP, revealed a difference between patients with CSCR and control subjects. The findings on the SD-OCT was similar to OCT-A response after HGT, further suggesting that hemodynamic stress, a physiological mechanism regulating the choroidal blood flow, may not be efficient in CSCR [2]. When there is an increase in BP as a result of physical or emotional stress, the sympathetic autoregulatory system causes vasoconstriction in order to prevent hyperperfusion in healthy subjects. In CSCR patients, autonomic dysfunction has been thought to result in choroidal hyperperfusion and secondary RPE dysfunction [20,21,22,37].

Our observation of a non-significant difference in CVI between patients with chronic CSCR and healthy controls at baseline are in agreement with data obtained by Agrawal et al. [38]. The MOPP, a pressure that depends on the mean arterial pressure and the IOP, determines blood flow in the choroidal vessels [20]. In this study, we have experimentally increased systemic BP and MOPP through an isometric HG exercise that produced active stimulation of the mechanoreceptors of the sympathetic system [39]. During the stress test in healthy controls, the DA of the choroid was significantly reduced while the CA and BA were not (Table 1 and Table 2). In healthy controls, when the MOPP increases, the choroid protects itself with a vasoconstriction of arterioles, which implies an increase in the dark region and reduction in the CVI on the tomographic structural images. In contrast, chronic CSCR patients had no statistically significant changes in either the CVI or the dark area (Figure 1 and Figure 2), suggesting vascular dysregulation in the choroid of CSCR patients, supporting the hypothesis of an impaired mechanism of choroidal blood flow regulation in the pathogenesis of CSCR [40,41,42,43]. Blood flow to the choriocapillaris (CC) is modulated by the choroid through its own mechanisms of regulation when MOPP changes [20,21,22]. Tittl et al. tested this property, measuring, by means of laser Doppler flowmetry, CC blood flow after a squatting isometric test (in order to increase BP and MOPP); in CSCR patients, this regulatory mechanism was found to be lacking [23].

A vigorous physical activity generating frequent hypertensive spikes has been recently reported as a risk factor for CSCR [24,25]. The results of our study highlight the hemodynamic instability in patients with CSCR. The increased BP due to emotional and physical stress situations tends to produce peripheral vascular hyperperfusion that may have long-term consequences.

Previously, choroidal response to stress tests have already been investigated using SD-OCT and enhanced depth imaging [44,45]. In healthy subjects, choroidal thickness does not significantly increase in response to systemic systolic BP spike, while Roybal et al. reported increased choroidal thickness as a response to BP rise in patients with CSCR and not in controls [45,46]. However, this study goes further in quantifying the change using a systemic method.

Mechanisms that might explain the inability of CSCR patients to regulate the CVI in response to physical effort and BP increase could be a local neural dysregulation or a dysfunction of the endothelial-related autoregulation [24]. Moreover, myogenic regulatory mechanisms may be involved, as they play a major role in the instantaneous vascular response to BP changes [40].

It is known that BP fluctuations and episodic hypertension are more harmful to target organs than stable hypertension [46]. Frequent heavy physical activities, particularly isometric activities which most increase the blood pressure, can greatly impact the systemic and ocular/choroidal circulation and therefore should be limited by CSCR patients [47]. Our data suggest that risk factors for CSCR may both have a significant impact on systemic and choroidal hemodynamics. Moreover, in accordance with other observations reported by one of our authors (F.C.P), lower IOP values may be correlated with CSCR [48]. A low IOP can contribute to an elevation in the OPP and increase the transmural pressure in the CC, which favors extravascular leakage of fluid. However, this study has potential limitations. The small sample size is this study’s primary drawback. Second, diurnal variations in pachychoroid diseases and even in normal eyes could have influenced the results of this study [29,49].

## 5. Conclusions

The purpose of our study was to quantitatively investigate the possible differences between chronic CSCR patients and healthy subjects in choroidal structural response to sudden changes in blood pressure. Future studies should explore the potential correlation between the degree of dysfunction, with related different degrees of structural choroidal changes, and the severity of the disease. More information from further studies could guide us in the choice of better therapeutic strategies in these particular cases. It might also be interesting to apply the model of choroidal vascular reactivity to induced hemodynamic stress to other pathologies included in the spectrum of pachychoroid disease [50].

## Figures and Tables

**Table 1:** Intraocular pressure and ocular perfusion pressure data in CSCR patients and healthy controls at baseline and during the isometric hand-grip exercise. CSCR: central serous chorioretinopathy; IOP: intraocular pressure; OPP: ocular perfusion pressure.

	CSCR Patients					Healthy Controls					*p*-Value CSCR vs. Controls Baseline	*p*-Value CSCR vs. Controls Under Stress
	Baseline		Under Stress		*p*-Value Paired Data	Baseline		Under Stress		*p*-Value Paired Data		
	Mean	SD	Mean	SD		Mean	SD	Mean	SD			
IOP	13.43	1.81	13.17	1.42	0.137	14.40	1.51	14.20	1.62	0.527	0.154	0.080
OPP	56.22	6.12	74.75	7.32	<0.05	50.07	3.58	67.89	3.66	<0.05	<0.05	<0.05

**Table 2:** Absolute and relative differences between baseline and under-stress values in terms of intraocular pressure and ocular perfusion pressure in CSCR patients and healthy controls. CSCR: central serous chorioretinopathy; IOP: intraocular pressure; OPP: ocular perfusion pressure.

	CSCR Patients				Healthy Controls			
	Absolute Difference (Δ)		Relative Difference (Δr)		Absolute Difference (Δ)		Relative Difference (Δr)	
	Mean	SD	Mean	SD	Mean	SD	Mean	SD
IOP	−0.267	1.015	−1.4%	7.6%	−0.200	1.033	−1.3%	7.2%
OPP	18.533	7.223	33.9%	15.2%	17.820	3.739	36.0%	9.0%

## Data Availability

The original contributions presented in the study are included in the article, further inquiries can be directed to the corresponding authors.
